# Supplementary figures and images for: Genome Sequences of Populus tremula Chloroplast and Mitochondrion: Implications for Holistic Poplar Breeding
Source: PLoS One. 2016 Jan 22;11(1):e0147209. doi: 10.1371/journal.pone.0147209 (PMC4723046; doi:10.1371/journal.pone.0147209)

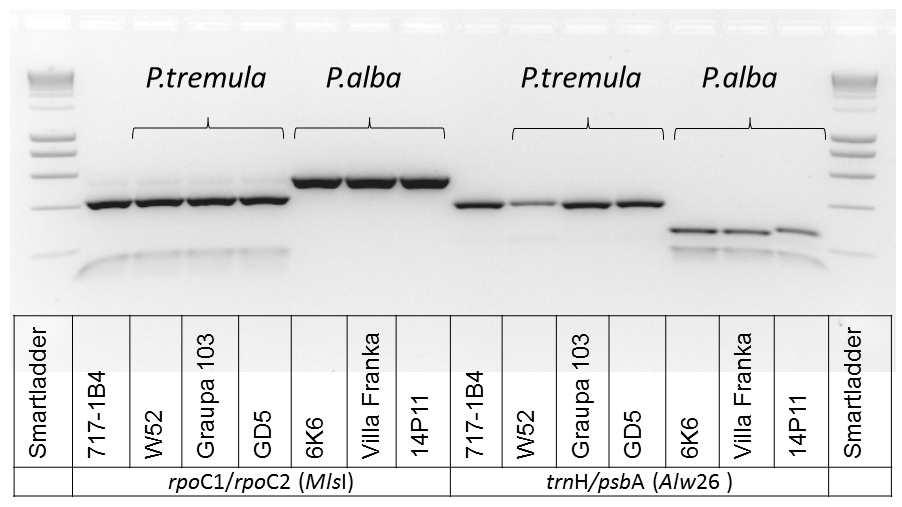

Supplement: S2 Appendix — Three other P. tremula individuals (W52, Graupa 103, GD5) and three P. alba individuals (6K6, Villa Franka, 14P11) were used as controls. (TIF) [file pone.0147209.s002.tif]

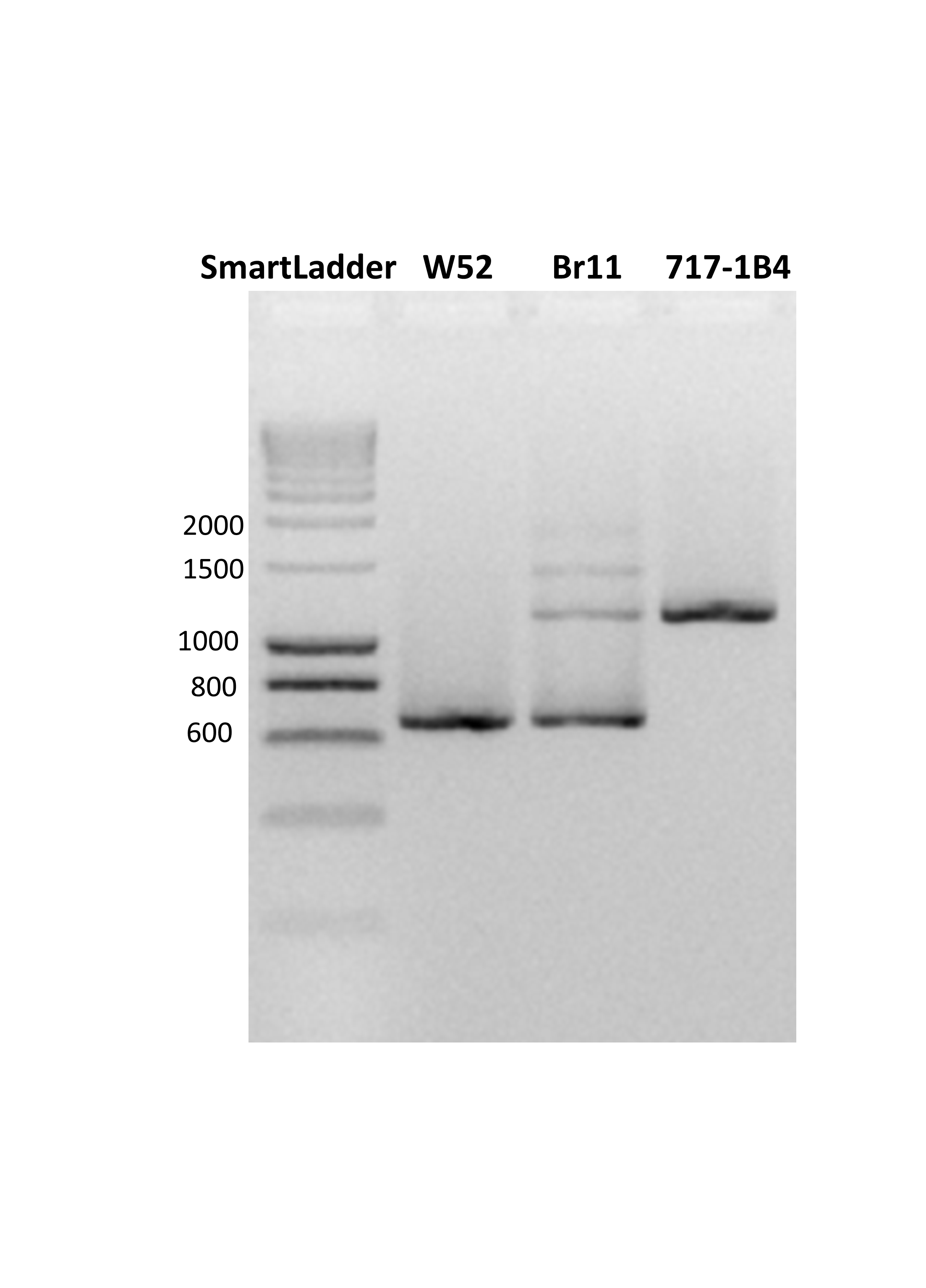

Supplement: S4 Appendix — DNA sequences of the primers “Ptre_IRA_SSC_for/rev” are given in S1 appendix. The theoretical lengths of the PCR products are 665 bp (W52) or 1126 bp (717-1B4), respectively, based on the related cpDNA sequences. (TIF) [file pone.0147209.s004.tif]

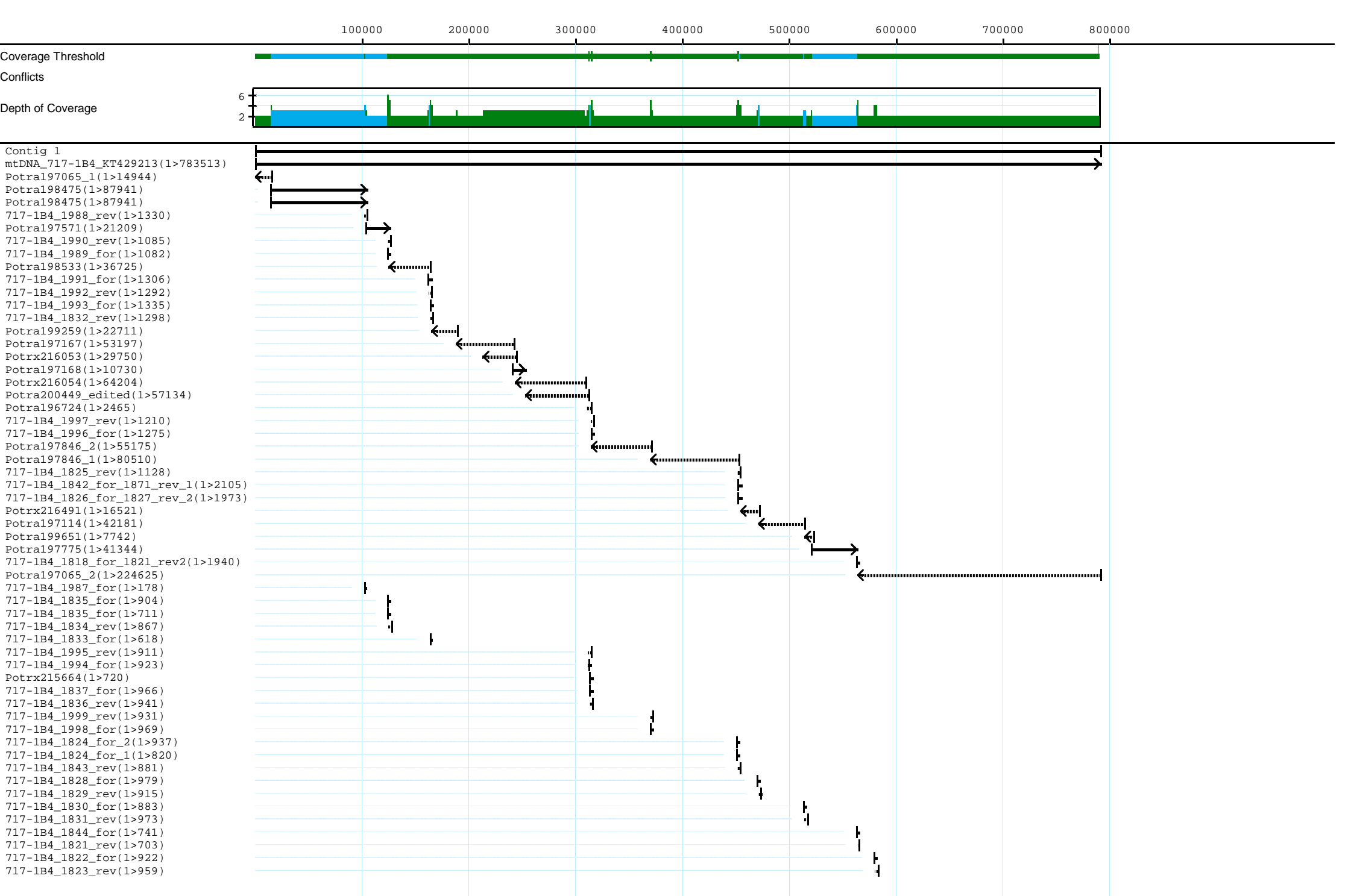

Supplement: S12 Appendix — The alignment was created by SeqMan Pro (v10.1.2. DNA Star, Madison, USA). The figure gives the alignment overview (“strategy view” of SeqMan Pro). Sanger sequences of PCR amplicons of 717-1B4 are named with the numbers of the forward/reverse primer, e.g. “717-1B4_1834”_for. P. tremula or P. tremula x P. tremuloides (T89) scaffolds selected from PopGenie are named with “Potra…” or “Ptrx…”, respectively. Forward and reverse Sanger sequences that did not overlap were combined and separated by respective N-stretches. (PDF) [file pone.0147209.s012.pdf]
