# Supplementary material for: Genome Sequences of Populus tremula Chloroplast and Mitochondrion: Implications for Holistic Poplar Breeding
Source: PLoS One. 2016 Jan 22;11(1):e0147209. doi: 10.1371/journal.pone.0147209 (PMC4723046; doi:10.1371/journal.pone.0147209)
Supplement: S3 Appendix — (DOCX) [file pone.0147209.s003.docx]

**Trimming and *de novo* assembly of the NGS data**
Initial quality control of the NGS reads (miSeq or IonTorrent) was performed with FastQC (http://www.bioinformatics.babraham.ac.uk/projects/fastqc/). Based on the FastQC reports all reads were trimmed using the trimming tool of the CLC GWB. All miSeq short reads containing adapter sequences (i.e. Illumina TrueSeq Universal Adapter, Index adapter I and TruSeq control sequences) were trimmed. Reads containing bases with a quality Phred score less than 20 were trimmed by setting the quality limit of the trimming tool to a value of 0.01. Ten nucleotides 3’ and 5’ of every read were removed and finally every read with a length of less than 100 bp was discarded. To reduce the amount of redundant data all overlapping read pairs were merged and duplicate reads were removed from the data set. *De novo* sequence assembly of the miSeq reads was performed with a preset word size of 29, length fraction of 0.9, similarity fraction of 0.95 and without the scaffolding option. The minimum and maximum read pair distances were set to 379 bp and 579 bp, respectively (based on the mean fragment size of the 717-1B4 genomic library). All remaining parameters were set to default (with back-mapping option per default).

All Ion Proton reads containing the adapter sequence (3'ATCACCGACTGCCCATAGAGAGGCTGAGAC5’) were trimmed. Reads containing bases with a quality Phred score less than 15 were trimmed by setting the quality limit of the trimming tool to a value of 0.03. Furthermore reads with a length less than 75 bp and duplicate reads were discarded. *De novo* sequence assembly of the trimmed reads was performed with a length fraction of 0.9 and a similarity fraction of 0.95. All remaining parameters were set to default.
