# Supplementary material for: Genome Sequences of Populus tremula Chloroplast and Mitochondrion: Implications for Holistic Poplar Breeding
Source: PLoS One. 2016 Jan 22;11(1):e0147209. doi: 10.1371/journal.pone.0147209 (PMC4723046; doi:10.1371/journal.pone.0147209)
Supplement: S8 Appendix — Identities (in %) of the cpDNA sequences in pairwise comparisons. (PDF) [file pone.0147209.s008.pdf]

		1	2	3	4	5	6	7	8	9
P_balsamifera	1		98.83	98.48	97.50	98.39	97.67	97.65	97.47	97.89
P_trichocarpa	2	98.83		99.39	97.74	98.67	98.05	98.09	97.95	98.34
P_euphratica	3	98.48	99.39		97.64	98.34	97.97	98.02	97.86	98.23
P_tremula_717-1B4	4	97.50	97.74	97.64		97.57	97.80	97.50	98.99	98.34
P_fremontii	5	98.39	98.67	98.34	97.57		97.57	97.59	97.46	97.83
P_yunnanensis	6	97.67	98.05	97.97	97.80	97.57		98.41	98.09	98.67
P_cathayana	7	97.65	98.09	98.02	97.50	97.59	98.41		97.82	98.33
P_tremula_W52	8	97.47	97.95	97.86	98.99	97.46	98.09	97.82		98.74
P_alba	9	97.89	98.34	98.23	98.34	97.83	98.67	98.33	98.74	
