# Supplementary material for: Genome Sequences of Populus tremula Chloroplast and Mitochondrion: Implications for Holistic Poplar Breeding
Source: PLoS One. 2016 Jan 22;11(1):e0147209. doi: 10.1371/journal.pone.0147209 (PMC4723046; doi:10.1371/journal.pone.0147209)
Supplement: S11 Appendix — This section represents the broader IRa-SSC linker region. (PDF) [file pone.0147209.s011.pdf]

```
                  114,620     114,640     114,660     114,680     114,700     114,720
P_balsamifera     GGAAAAGAAA GAATATCCTT TACGTATCCA CCCAGTTTGT CAACTTTTTT GGAAATGATA CAAAGAAAGA TGTCTCTGTT CACAACAGAA AAACTCTCCT CTGATGAATT GTATAATCAT 112219
P_trichocarpa     GGAAAAGAAA GAATATCCTT TACGTATCCA CCCAGTTTGT CAACTTTTTT GGAAATGATA CAAAGAAAGA TGTCTCTGTT CACAACAGAA AAACTCTCCT CTGATGAATT GTATAATCAT 112418
P_euphratica      GGAAAAGAAA GAATATCCTT TACGTATCCA CCCAGTTTGT CAACTTTTTT GGAAATGATA CAAAGAAAGA TGTCTCTGTT CACAACAGAA AAACTCTCCT CTGATGAATT GTATAATCAT 112170
P_tremula_717-1B4 GGAAAAGAAA GAATATCCTT TACGTATCCA CCCAGTTTGT CAACTTTTTT GGAAATGATA CAAAGAAAGA TGTCTCTGTT CACAACAGAA AAACTCTCCT CTGATGAATT GTATAATCAT 111669
P_fremontii       GGAAAAGAAA GAATATCCTT TACGTATCCA CCCAGTTTGT CAACTTTTTT GGAAATGATA CAAAGAAAGA TGTCTCTGTT CACAACAGAA AAACTCTCCT CTGATGAATT GTATAATCAT 112728
P_yunnanensis     GGAAAAGAAA GAATATCCTT TACGTATCCA CCCAGTTTGT CAACTTTTTT GGAAATGATA CAAAGAAAGA TGTCTCTGTT CACAACAGAA AAACTCTCCT CTGATGAATT GTATAATCAT 111236
P_cathayana       GGAAAAGAAA GAATATCCTT TACGTATCCA CCCAGTTTGT CAACTTTTTT GGAAATGATA CAAAGAAAGA TGTCTCTGTT CACAACAGAA AAACTCTCCT CTGATGAATT GTATAATCAT 111091
P_tremula_W52     GGAAAAGAAA GAATATCCTT TACGTATCCA CCCAGTTTGT CAACTTTTTT GGAAATGATA CAAAGAAAGA TGTCTCTGTT CACAACAGAA AAACTCTCCT CTGATGAATT GTATAATCAT 111616
P_alba            GGAAAAGAAA GAATATCCTT TACGTATCCA CCCAGTTTGT CAACTTTTTT GGAAATGATA CAAAGAAAGA TGTCTCTGTT CACAACAGAA AAACTCTCCT CTGATGAATT GTATAATCAT 111917
Consensus         GGAAAAGAAA GAATATCCTT TACGTATCCA CCCAGTTTGT CAACTTTTTT GGAAATGATA CAAAGAAAGA TGTCTCTGTT CACAACAGAA AAACTCTCCT CTGATGAATT GTATAATCAT
```

```
                  114,740     114,760     114,780     114,800     114,820     114,840
P_balsamifera     TGGAATTATA ACAATGAACA AAAAAAGAAA AACCTAAGTA ACGAGTTTAT AAATAGAGTA GAAGTTCTAG ATAAGGGGTT TCGTGTTCTG AATATACTCG AAAAAAAGAC TAGATTGTGT 112339
P_trichocarpa     TGGAATTATA ACAATGAACA AAAAAAGAAA AACCTAAGTA ACGAGTTTAT AAATAGAGTA GAAGTTCTAG ATAAGGGGTT TCGTGTTCTG AATATACTCG AAAAAAAGAC TAGATTGTGT 112538
P_euphratica      TGGAATTATA ACAATGAACA AAAAAAGAAA AACCTAAGTA ACGAGTTTAT AAATAGAGTA GAAGTTCTAG ATAAGGGGTT TCGTGTTCTG AATATACTCG AAAAAAAGAC TAGATTGTGT 112290
P_tremula_717-1B4 TGGAATTATA ACAATGAACA AAAAAAGAAA AACCTAAGTA ACGAGTTTAT AAATAGAGTA GAAGTTCTAG ATAAGGGGTT TCGTGTTCTG AATATACTCG AAAAAAAGAC TAGATTGTGT 111789
P_fremontii       TGGAATTATA ACAATGAACA AAAAAAGAAA AACCTAAGTA ACGAGTTTAT AAATAGAGTA GAAGTTCTAG ATAAGGGGTT TCGTGTTCTG AATATACTCG AAAAAAAGAC TAGATTGTGT 112848
P_yunnanensis     TGGAATTATA ACAATGAACA AAAAAAGAAA AACCTAAGTA ACGAGTTTAT AAATAGAGTA GAAGTTCTAG ATAAGGGGTT TCGTGTTCTG AATATACTCG AAAAAAAGAC TAGATTGTGT 111356
P_cathayana       TGGAATTATA ACAATGAACA AAAAAAGAAA AACCTAAGTA ACGAGTTTAT AAATAGAGTA GAAGTTCTAG ATAAGGGGTT TCGTGTTCTG AATATACTCG AAAAAAAGAC TAGATTGTGT 111211
P_tremula_W52     TGGAATTATA ACAATGAACA AAAAAAGAAA AACCTAAGTA ACGAGTTTAT AAATAGAGTA GAAGTTCTAG ATAAGGGGTT TCGTGTTCTG AATATACTCG AAAAAAAGAC TAGATTGTGT 111736
P_alba            TGGAATTATA ACAATGAACA AAAAAAGAAA AACCTAAGTA ACGAGTTTAT AAATAGAGTA GAAGTTCTAG ATAAGGGGTT TCGTGTTCTG AATATACTCG AAAAAAAGAC TAGATTGTGT 112037
Consensus         TGGAATTATA ACAATGAACA AAAAAAGAAA AACCTAAGTA ACGAGTTTAT AAATAGAGTA GAAGTTCTAG ATAAGGGGTT TCGTGTTCTG AATATACTCG AAAAAAAGAC TAGATTGTGT
```

```
                  114,860     114,880     114,900     114,920     114,940     114,960
P_balsamifera     AATGATGAAA CTAAAAAAGA ATACTTACCT AAAATATATG ATCCTTTTTT GAGTGGGCTC TATCGTGGAA AAGTCAATTT TTTTTTTTCA CCCTCAATCA TAAATGAAAT TTCCATAAAA 112459
P_trichocarpa     AATGATGAAA CTAAAAAAGA ATACTTACCT AAAATATATG ATCCTTTTTT GAGTGGGCTC TATCGTGGAA AAGTCAATTT TTTTTTTTCA CCCTCAATCA TAAATGAAAT TTCCATAAAA 112658
P_euphratica      AATGATGAAA CTAAAAAAGA ATACTTACCT AAAATATATG ATCCTTTTTT GAGTGGGCTC TATCGTGGAA AAGTCAATTT TTTTTTTTCA CCCTCAATCA TAAATGAAAT TTCCATAAAA 112410
P_tremula_717-1B4 AATGATGAAA CTAAAAAAGA ATACTTACCT AAAATATATG ATCCTTTTTT GAGTGGGCTC TATCGTGGAA AAGTCAATTT TTTTTTTTCA CCCTCAATCA TAAATGAAAT TTCCATAAAA 111909
P_fremontii       AATGATGAAA CTAAAAAAGA ATACTTACCT AAAATATATG ATCCTTTTTT GAGTGGGCTC TATCGTGGAA AAGTCAATTT TTTTTTTTCA CCCTCAATCA TAAATGAAAT TTCCATAAAA 112968
P_yunnanensis     AATGATGAAA CTAAAAAAGA ATACTTACCT AAAATATATG ATCCTTTTTT GAGTGGGCTC TATCGTGGAA AAGTCAATTT TTTTTTTTCA CCCTCAATCA TAAATGAAAT TTCCATAAAA 111476
P_cathayana       AATGATGAAA CTAAAAAAGA ATACTTACCT AAAATATATG ATCCTTTTTT GAGTGGGCTC TATCGTGGAA AAGTCAATTT TTTTTTTTCA CCCTCAATCA TAAATGAAAT TTCCATAAAA 111331
P_tremula_W52     AATGATGAAA CTAAAAAAGA ATACTTACCT AAAATATATG ATCCTTTTTT GAGTGGGCTC TATCGTGGAA AAGTCAATTT TTTTTTTTCA CCCTCAATCA TAAATGAAAT TTCCATAAAA 111856
P_alba            AATGATGAAA CTAAAAAAGA ATACTTACCT AAAATATATG ATCCTTTTTT GAGTGGGCTC TATCGTGGAA AAGTCAATTT TTTTTTTTCA CCCTCAATCA TAAATGAAAT TTCCATAAAA 112157
Consensus         AATGATGAAA CTAAAAAAGA ATACTTACCT AAAATATATG ATCCTTTTTT GAGTGGGCTC TATCGTGGAA AAGTCAATTT TTTTTTTTCA CCCTCAATCA TAAATGAAAT TTCCATAAAA
```

```
                  114,980     115,000     115,020     115,040     115,060     115,080
P_balsamifera     AATTACATAG AGATGCTTTG GATAAATAAA ATTTATCTTA TCCTTCTTAT TTCTAATTAT CAAGAATTTG AACCAAAAAT GGAGGATCGA ATAAGAATTT TAAAATTTTT ATTTGATGCA 112579
P_trichocarpa     AATTACATAG AGATGCTTTG GATAAATAAA ATTTATCTTA TCCTTCTTAT TTCTAATTAT CAAGAATTTG AACCAAAAAT GGAGGATCGA ATAAGAATTT TAAAATTTTT ATTTGATGCA 112778
P_euphratica      AATTACATAG AGATGCTTTG GATAAATAAA ATTTATCTTA TCCTTCTTAT TTCTAATTAT CAAGAATTTG AACCAAAAAT GGAGGATCGA ATAAGAATTT TAAAATTTTT ATTTGATGCA 112530
P_tremula_717-1B4 AATTACATAG AGATGCTTTG GATAAATAAA ATTTATCTTA TCCTTCTTAT TTCTAATTAT CAAGAATTTG AACCAAAAAT GGAGGATCGA ATAAGAATTT TAAAATTTTT ATTTGATGCA 112029
P_fremontii       AATTACATAG AGATGCTTTG GATAAATAAA ATTTATCTTA TCCTTCTTAT TTCTAATTAT CAAGAATTTG AACCAAAAAT GGAGGATCGA ATAAGAATTT TAAAATTTTT ATTTGATGCA 113088
P_yunnanensis     AATTACATAG AGATGCTTTG GATAAATAAA ATTTATCTTA TCCTTCTTAT TTCTAATTAT CAAGAATTTG AACCAAAAAT GGAGGATCGA ATAAGAATTT TAAAATTTTT ATTTG----- 111591
P_cathayana       AATTACATAG AGATGCTTTG GATAAATAAA ATTTATCTTA TCCTTCTTAT TTCTAATTAT CAAGAATTTG AACCAAAAAT GGAGGATCGA ATAAGAATTT TAAAAAAACC- ATTTG----- 111445
P_tremula_W52     AATTACATAG AGATGCTTTG GATAAATAAA ATTTATCTTA TCCTTCTTAT TTCTAATTAT CAAGAATTTG AACCAAAAAT GGAGGATCGA ATAAGAATTT TAAAATTTTT ATTTGATG-- 111974
P_alba            AATTACATAG AGATGCTTTG GATAAATAAA ATTTATCTTA TCCTTCTTAT TTCTAATTAT CAAGAATTTG AACCAAAAAT GGAGGATCGA ATAAGAATTT TAAAATTTTT ATTTGATG-- 112275
Consensus         AATTACATAG AGATGCTTTG GATAAATAAA ATTTATCTTA TCCTTCTTAT TTCTAATTAT CAAGAATTTG AACCAAAAAT GGAGGATCGA ATAAGAATTT TAAAATTTTT ATTTGATGCA
```

```
                  115,100     115,120     115,140     115,160     115,180     115,200
P_balsamifera     ATTATAGTGG ATTCCAAGAA TAAAAAAATT GTAAAAAAAT CTACAGAAAT AAAAAAAGAA ATAAGTAAAC AAATTCCTCG ATGGTCATAC AAATTAATCG ACGATTTGGA ACAACAAGAG 112699
P_trichocarpa     ATT------- ---------- ---------- ---------- ---------- ---------- ---------- ---------- ---------- ---------- ---------- ---------- 112781
P_euphratica      ATT------- ---------- ---------- ---------- ---------- ---------- ---------- ---------- ---------- ---------- ---------- ---------- 112533
P_tremula_717-1B4 ATTATAGTGG ATTCCAAGAA TAAAAAAATT GTAAAAAAAT CTACAGAAAT AAAAAAAGAA ATAAGTAAAC AAATTCCTCG ATGGTCATAC AAATTAATCG ACGATTTGGA ACAACAAGAG 112149
P_fremontii       ATTGTAGTGG ATTCAAAGAA TAAAAAAATT GTAAAAAAAT CTACAGAAAT AAAAAAAGAA ATAAGTAAAC AAATTCCTCG ATGGTCATAC AAATTAATCG ACGATTTGGA ACAACAAGAG 113208
P_yunnanensis     ---------- ---------- ---------- ---------- ---------- ---------- ---------- ---------- ---------- ---------- ---------- ---------- 111591
P_cathayana       ---------- ---------- ---------- ---------- ---------- ---------- ---------- ---------- ---------- ---------- ---------- ---------- 111445
P_tremula_W52     ---------- ---------- ---------- ---------- ---------- ---------- ---------- ---------- ---------- ---------- ---------- ---------- 111974
P_alba            ---------- ---------- ---------- ---------- ---------- ---------- ---------- ---------- ---------- ---------- ---------- ---------- 112275
Consensus         ATT------- ---------- ---------- ---------- ---------- ---------- ---------- ---------- ---------- ---------- ---------- ----------
```

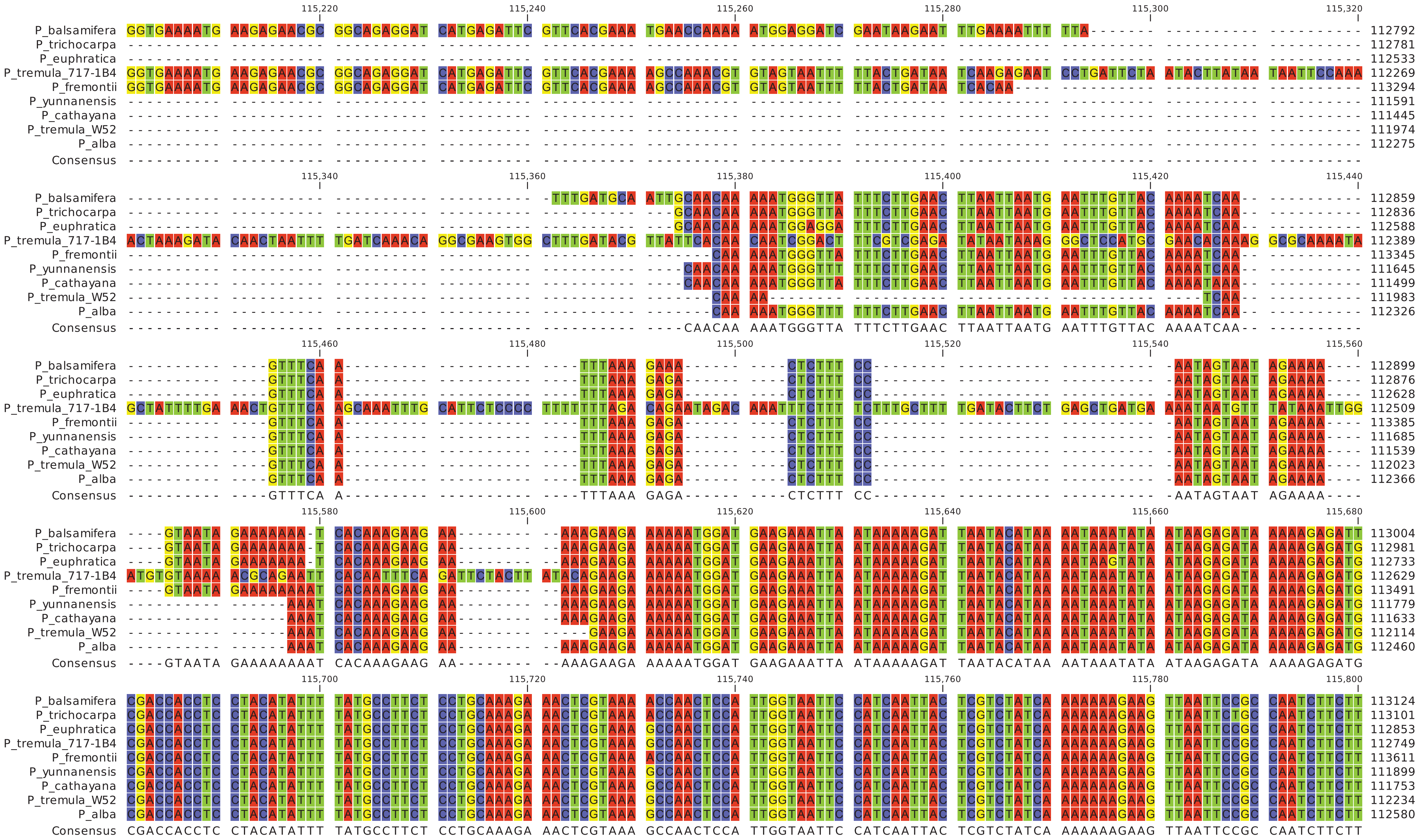
P_balsamifera
P_trichocarpa
P_euphratica
P_tremula_717-1B4
P_fremontii
P_yunnanensis
P_cathayana
P_tremula_W52
P_alba
Consensus
115,220
115,240
115,260
115,280
115,300
115,320
115,340
115,360
115,380
115,400
115,420
115,440
115,460
115,480
115,500
115,520
115,540
115,560
115,580
115,600
115,620
115,640
115,660
115,680
115,700
115,720
115,740
115,760
115,780
115,800
